# The influence of individual, peer, and family factors on the educational aspirations of adolescents in rural China

**DOI:** 10.1007/s11218-023-09765-3

**Published:** 2023-01-27

**Authors:** Xiaodi Chen, Jennifer L. Allen, Therese Hesketh

**Affiliations:** 1 Institute for Global Health, University College London, 30 Guilford Street, London, WC1N 1EH UK; 2 Department of Psychology, University of Bath, Bath, UK; 3 Center for Global Health, Zhejiang University School of Medicine, Hangzhou, China

**Keywords:** Educational aspirations, Parent–child relationship, Peer relationships, Rural adolescents, China

## Abstract

Educational aspirations are an important predictor of academic outcomes. While there has been considerable research on educational aspirations in the West, there has been little research in East Asia, and the investigation of factors influencing adolescent aspirations has been neglected, particularly in rural areas. Drawing on ecological systems theory and social cognitive career theory, this study investigated the associations between educational aspirations and factors at the individual, peer, and family levels among rural Chinese adolescents. A total of 606 students (*M*_age_ = 14.85 years; 50% boys) from a rural town in Central China completed questionnaires assessing their educational aspirations, individual factors (academic performance, academic self-perception, academic self-regulation, attitudes toward teachers, and goal valuation), and contextual factors (family socioeconomic status, parent and peer relationship quality, and parental and close friends’ aspirations). Individual factors and aspirations of others had significant direct effects on adolescents’ educational aspirations, while parent and peer attachments were significantly related to educational aspirations via individual factors. Family socioeconomic status was not significantly related to adolescents’ educational aspirations. The findings highlight the importance of individual factors as mechanisms explaining the link between contextual factors and rural Chinese adolescents’ educational aspirations. Our results suggest that interventions can be designed to increase and maintain the aspirations of rural Chinese youth by targeting multiple domains of influence.

## Introduction

Educational aspirations are idealistic values that reflect the educational attainment that one hopes and desires to achieve (Khattab, 2015). They have been shown to have an important influence on adolescent outcomes, including school performance, occupational attainment, and adjustment (Boxer et al., 2011). Therefore, it is necessary to examine the factors that influence the goals that students have for their educational careers. According to Bronfenbrenner’s (1979) ecological theory of child development, “an accurate and comprehensive understanding of any child’s developmental issues necessarily includes recognition of the individual and contextual influences that shape their beliefs, attitudes, and behaviors” (Nichols et al., 2010, p. 28). Consistent with this, current theoretical models highlight the importance of personal, family, and school domains in determining adolescents’ educational aspirations (e.g., Garg et al., 2002). However, little research spanning these multiple domains of influence has been conducted in non-Western nations (Leung et al., 2017). It is important to investigate the educational aspirations of young people in East Asia, given the cultural differences in social norms and education systems. The value of education is highly emphasized in China, and for many young people in rural China, education is seen as a route out of poverty and a pathway to upward social mobility (Wu & Treiman, 2007). As such, investigating the factors that influence adolescents’ educational aspirations at multiple levels is relevant to developing effective policies and interventions to increase the likelihood of successful outcomes for young people in rural China.

### Adolescents in rural China and education

In China, the nine years of compulsory education are divided into six years of primary education (grades 1–6) and three years of middle school (grades 7–9). Upon finishing middle school, a typical student progresses to either a regular high school or a vocational and technical school based on his or her scores on the National High School Entrance Examination. Those unable or unwilling to attend regular high school can enroll in vocational schools; such students accounted for 39.5% of students in 2019 (Chinese Ministry of Education, 2020). Regular high schools focus on university preparation, whereas vocational schools focus on vocational skills. However, vocational education has a lower status and less prestige than regular (i.e., academic) education in China; thus, many students and their parents see vocational schools as a last resort (Hansen & Woronov, 2013).

Residence in an urban or rural area is a critical factor in determining one’s educational opportunities in China, including progression to a regular high school and university (Knight & Li, 1996). Chinese adolescents growing up in rural areas face many challenges that can adversely impact their education due to limited educational resources and economic pressures (Li et al., 2015; Wang, 2014). For example, by examining the China University Student Survey, Li et al. (2015) found that rural youth from poor counties were seven and 11 times less likely to get into any university and first-rate universities than urban youth, respectively. To reduce the rural‒urban inequality in regard to educational opportunities, the Chinese government has boosted its efforts to implement a series of policies and programs related to developing higher education since 2000, resulting in a rapid expansion of higher education. Over the past 20 years, the number of government-funded higher education institutions (colleges and universities) increased from 1100 to 2738 (State Statistics Bureau, 2021). Given this rapid expansion, the act of increasing or maintaining rural students’ desire to pursue higher education is receiving more attention from educators and policy-makers (Wu, 2020).

Early adolescence is a developmental period during which young people begin to think about their future (Massey et al., 2008). Although previous studies have examined the factors influencing the educational goals of young people in China (Fang, 2016; Guo et al., 2014), the aspirations of adolescents in rural areas have largely been ignored. Therefore, the current study will address this gap by examining the factors that influence the educational aspirations of rural adolescents who may be educationally disadvantaged.

### Theoretical frameworks of educational aspirations

Bronfenbrenner’s (1979) ecological systems framework has been applied to a broad range of developmental issues in education, including educational aspirations, school engagement, and academic achievement (e.g., Dotterer & Lowe, 2011; Hampden-Thompson & Galindo, 2017; Nichols et al., 2010). Ecological systems theory emphasizes that children grow up in multiple interacting nested systems, with their development influenced by people and experiences at multiple levels of these systems, from microsystems such as parents and peers to macrosystems such as cultural contexts (Bronfenbrenner, 1979). Understanding child‒context interactions is central to Bronfenbrenner’s ecological model of child development (Garbarino & Ganzel, 2000). Consistent with this ecological systems perspective, previous studies have found that adolescents’ characteristics interact with immediate context influences, such as family and school, to influence their educational aspirations (e.g., Nichols et al., 2010).

In addition, social cognitive career theory (SCCT; Lent et al., 1994, 2000) extends and adapts Bandura’s (1976, 2001) general social cognitive theory to explain an individual’ pursuit of occupational and educational goals. SSCT holds that personal (e.g., ethnicity) and contextual factors (e.g., family background, educational experiences) create unique learning experiences that facilitate the development of personal attributes related to internal cognitive and affective states (termed cognitive-person variables), such as self-efficacy and goal valuation, which in turn influence an individual’s academic and career-related goals and actions (Johnson, 2013; Lent et al., 2000). SCCT highlights cognitive-person variables and their interplay with contextual factors during the process of goal setting (Lent et al., 2000). In the present study, we extended SCCT to the educational domain, in line with previous studies (e.g., Curtin et al., 2016; Kantamneni et al., 2018).

Previous studies have investigated how individual child, family, and peer factors are independently associated with Chinese adolescents’ aspirations (e.g., Guo et al., 2014). However, to our knowledge, no empirical research in China has examined how factors from multiple domains simultaneously influence adolescents’ educational aspirations. Furthermore, given that most previous research on factors influencing adolescents’ educational aspirations is limited to Western samples, it is unclear how these multiple domains of influence apply to rural Chinese youth. Therefore, guided by Bronfenbrenner’s (1979) ecological systems framework and SCCT, this study addressed the literature gap by investigating the influence of individual (sociocognitive process) and contextual factors (parents, peers) on adolescents’ educational aspirations in rural Chinese middle school students. As SCCT posits that contextual influences on people’s educational and occupational choices are based on sociocognitive processes (Lent et al., 2000) and in light of previous findings (e.g., Curtin et al., 2016; Ojeda & Flores, 2008), we hypothesized that contextual factors would have a significant indirect effect on adolescents’ educational aspirations via cognitive-person factors.

### Factors associated with educational aspirations

Family socioeconomic status (SES) is an important factor influencing adolescents’ educational aspirations. In the Western context, Boxer et al. (2011) found that students from socially disadvantaged backgrounds are often aware of the barriers they face in pursuing academic success; thus, these students are more likely to disengage from education. Teachman and Paasch (1998) also found that adolescents from low-SES backgrounds in the United States of America (USA) are less likely to view attending higher education institutions as achievable compared with students from wealthier families with a similar level of academic ability. However, the situation is different among Chinese youth. Jiang (2011) found that college students from disadvantaged families have higher educational aspirations than their counterparts from high-SES backgrounds. Zhang (2016) found that Chinese adolescents aspire to attend university regardless of family SES. Confucian traditions strongly emphasize the acquisition of knowledge and the importance of education; thus, achieving educational achievement is a deeply rooted cultural belief among Chinese people (Fu et al., 2016). This belief in the value of education to achieve upward social mobility and enhance the reputation of one’s family further leads rural families to place a high value on education despite barriers to their ability to access good quality education (Luo et al., 2018). Furthermore, adolescents in the current study were from Hubei Province, an economically deprived inland province where education is seen as the only way for rural students to escape poverty (Sier et al., 2021). Thus, there appear to be different cultural perspectives on the use of educational attainment as a means by which to achieve wealth and social status. Therefore, we expected that a lower family SES would be associated with a higher evaluation of educational goals, which would in turn predict higher educational aspirations among the adolescents participating in the current study.

Parents’ educational aspirations for their children have been identified as having an important influence on children’s educational aspirations. Adolescents are more likely to complete compulsory education and continue to pursue higher education if their parents hold high educational aspirations for them (Glick & White, 2004). Consistent with findings in Western nations, parental aspirations for their children’s education are a key contributor to the academic attainment and aspirations of Chinese middle school students (Guo, 2014; Li, 2004). The pursuit of upward mobility is particularly important for parents from rural areas and thus strongly impacts the aspirations they set for their children (Wu & Treiman, 2007). Such parents tend to have a strong belief in the importance of education and hold high educational aspirations for their children (Koo et al., 2012). Moreover, traditional Confucian thinking values filial piety, with children expected to obey and respect their parents and to comply with their parents’ aspirations (Luo et al., 2013). Therefore, parents’ aspirations are likely to be an important factor in shaping the educational aspirations of rural Chinese adolescents.

The aspirations of others in adolescents’ peer and friendship groups are another factor that may influence educational aspirations, with peer relationships and social conformity coming into prominence during early adolescence. Adolescents tend to show similar levels of school-related adjustment to others in their peer group, including school performance and academic self-perception (Chen et al., 2003; Ryan, 2001). During the early years of high school, adolescents start to think about their future education and career; therefore, adolescents often discuss their views and aspirations with their peers (Eccles et al., 2004; Kiuru et al., 2007). Members of the same peer groups have been found to share similar educational goals, whether examined as a friendship pair (Hallinan & Williams, 1990) or as self-nominated friendship groups (Epstein, 1983). Chinese culture is collectivistic; thus, students are strongly influenced by their perceived views of others in their peer group (Liu & Chen, 2003). Accordingly, to fully understand rural adolescents’ aspirations, it is important to consider the aspirations of their parents and peers in parallel.

Connectedness and interdependence are given more attention in collective cultures such as China. It is well established that the quality of parent and peer relationships has a substantial impact on the academic outcomes of Chinese adolescents (e.g., Leung et al., 2017; Li et al., 2022). For example, Leung et al. (2017) found that Chinese adolescents who perceive their relationship with their parents as close and supportive have higher levels of self-confidence, an optimistic attitude toward their future, and invest more time and effort into reaching their goals. In the school setting, high-quality peer relationships contribute to the formation of a psychological attachment to school (Frostick et al., 2016). Adolescents are also more willing and confident in planning their educational future when they feel more attached to their schools (Wong et al., 2019). These findings, therefore, provide a foundation for the present study to explore the role of parents and peer relationship quality in influencing rural adolescents’ educational aspirations.

Regarding individual factors, we focused on cognitive-person variables in the school domain. There is substantial evidence for a relationship between academic achievement and educational aspirations (e.g., Garg et al., 2002; Mau & Bikos, 2000). Adolescents learn about their competence through feedback on their academic performance and form their educational aspirations based on this process (Garg et al., 2002). High-achieving students are more likely to have a better academic self-concept and greater confidence in their likelihood of success than low-achieving students (Pajares & Schunk, 2002; Strayhorn, 2009). In addition, adolescents’ educational aspirations are strongly related to their beliefs about academic-oriented goals (Kirk et al., 2012). Viewing the pursuit of knowledge as a moral virtue, mostly with roots in Confucianism, coupled with the expectations of a high-income return from completing further education, has led Chinese adolescents to highly value academic achievement, which often leads to higher educational aspirations (Ng & Wei, 2020). Other individual factors identified as important for educational aspirations include academic self-perception and self-regulation. Kirk et al. (2012) defined academic self-perception as students’ understanding and perception of their competence at school. Academic self-perception plays an important role in decision-making and goal setting concerning one’s education and career. Students with a positive academic self-perception are more likely than their peers with a negative academic self-perception to study harder and maintain their academic motivation (Jaiswal & Choudhuri, 2017). There is also evidence that without specific study strategies, adolescents find educational goals difficult to attain (Oyserman et al., 2004). Many adolescents set high academic goals but lack the self-regulated learning strategies (e.g., self-initiated actions to reach goals and time management) that facilitate this process (Lee & Oyserman, 2007). However, previous studies have overlooked the potential influence of academic self-regulation on educational aspirations (e.g., Garg et al., 2002; Rottinghaus et al., 2002). In the present study, we expected individual factors (i.e., attributes related to adolescents’ internal cognitive and affective states in the school domain), including perceived academic achievement, academic self-perception, goal valuation, academic self-regulation, and attitudes toward teachers, to have a direct influence on educational aspirations. Consistent with theory (Bronfenbrenner, 1979; Lent et al., 2000), we hypothesized that these individual factors would also be influenced by contextual factors such as parents’ aspirations and parent and peer relationships.

### The present study

In this study, we sought to assess the educational aspirations of adolescents living in rural China and predicted that they would have high aspirations. Consistent with ecological systems theory, SCCT, and previous research on educational goals in China (e.g., Wu et al., 2018; Zhang et al., 2016), we also predicted that low family SES, higher parental and peer educational aspirations, and good quality parent and peer relationships would be significantly associated with higher educational aspirations among rural adolescents and that these associations would work indirectly through individual factors (academic performance, academic self-perception, academic self-regulation, attitudes toward teachers, and goal valuation).

## Method

### Participants

The current study was conducted in Songzi, a poor rural county in Hubei Province in Central China. As a major agricultural province in the central region, Hubei Province has a large rural population (40.7% of the total population) and is considered to represent a wide cross-section of China’s rural areas (Li et al., 2020), making it a very suitable setting for studying rural youth in China. Songzi has 17 townships, and participants were recruited from Nanhai town, which has a total population of 57,500 (81.9% are rural residents) and two public middle schools (Peng, 2017). All students in grade 9 at these two schools during the 2019–2020 school year were invited to participate in the study. In China, this is the last year of compulsory education; therefore, it is a time when students need to consider their educational future. The original study sample consisted of 721 students. However, 95 students did not complete the questionnaires, while another 20 declined to participate, giving an overall participation rate of 84%. The final sample of 606 students consisted of 303 boys and 303 girls aged 13 to 16 years (*M*_age_ = 14.85 years, *SD* = 0.59). Participant demographic characteristics are presented in Table 1.

**Table 1:** Demographic characteristics of the sample (N = 606)

Variables	*%* (*N*)
Adolescent gender	
Male	50.0 (303)
Female	50.0 (303)
Only child family	
Yes	55.6 (337)
No	44.4 (269)
Mother’s educational level	
Primary school/below	18.0 (109)
Middle school/Vocational school	54.6 (331)
High school/above	14.5 (88)
Father’s educational level	
Primary school/below	14.2 (86)
Middle school/Vocational school	53.0 (321)
High school/above	19.5 (118)
Parents’ marital status	
Married	85.8 (520)
Divorced	14.2 (86)
Family wealth	
Low	44.9 (272)
Middle	40.1 (243)
High	15.0 (91)

### Procedure

Following receipt of ethical approval from the university ethics board, the head teachers and teaching staff at the two schools were approached, and their approval of the study was obtained. Data collection was conducted in two schools in June 2020. Adolescents with personal and parental (or caregiver) written informed consent participated in the study. Students completed the survey independently in their regular classroom under the supervision of a researcher who was available to answer questions throughout the session. The survey took approximately 40 min to complete.

### Measures

#### Demographics

Adolescents reported their age, gender, one-child family status, and parental marital status.

#### Educational aspirations

Adolescents reported, “What is the highest level of education that you wish to achieve?”. Responses were recorded on a 6-point Likert scale (1 = *completion of middle school*, 2 = *vocational school*, 3 = *high school*, 4 = *bachelor’s degree*, 5 = *master’s degree*, and 6 = *doctoral degree*). This item was based on similar measures that have been widely used in prior research on educational aspirations and achievement in both Western and Chinese contexts (e.g., Boxer et al., 2011; Fang, 2016; Leung et al., 2017).

#### Family SES

SES was assessed by asking adolescents to indicate the highest level of education their mother and father had attained (1 = *lower than elementary school*, 2 = *completion of elementary school*, 3 = *completion of middle school*, 4 = *completion of vocational school*, 5 = *completion of high school*, 6 = *higher than high school*, 7 = *I don’t know*). Adolescents also completed the Family Affluence Scale (FAS-II; Currie et al., 1997), which includes four items assessing universal aspects of family affluence (e.g., "Does your family own a car, van or truck?"). Responses were recorded on a 3-point scale (0 = *none (no)*, 1 = *once (one)*, 2 = *more than once (one)*, except that one item assessing ownership of the bedroom was coded as *yes* (= 1) and *no* (= 0). Three groups were formed based on the composite FAS score: low (score = 0‒3), medium (score = 4‒5), and high (score = 6‒7). The Chinese version of the FAS-II has good reliability and validity (e.g., Liu et al., 2012). The mean inter-item correlation for the four-item FAS-II scores in the current study was 0.20, suggesting good reliability (Piedmont, 2014). Parent education level was combined with FAS-II scores to create the measures of family SES.

#### Perceived academic performance

Perceived academic performance was assessed by asking each student to indicate “Which of the following best describes your academic performance in class this year?”. Responses were recorded on a 5-point scale (1 = *low, ranked lower than 50*, 2 = *low-middle, ranking 30‒50*, 3 = *middle, ranking 20‒30*, 4 = *upper-middle, ranking 10‒20*, and 5 = *top, ranking top 10*). The academic ranking has shown good validity as an indicator of academic performance in prior studies (e.g., Gao et al., 2020; Zhao et al., 2017).

#### Individual factors in the school domain

These factors were assessed using the School Attitude Assessment Survey-Revised (SAAS-R; McCoach & Siegle, 2003). The SAAS-R consists of four scales assessing attitudes toward teachers (7 items; e.g., “I like my teachers.”), goal valuation (6 items; e.g., “I want to do my best in school.”), academic self-regulation (10 items; e.g., “I complete my schoolwork regularly.”), and academic self-perceptions (7 items; e.g., “I am intelligent.”). Responses were recorded on a 7-point scale (1 = *disagree very strongly* to 7 = *agree very strongly*). Prior research using the SAAS-R has demonstrated its reliability and validity in East Asian samples (e.g., Chong et al., 2018). The alphas in the current study ranged from 0.87 to 0.93.

#### Adolescents’ perceived parental aspirations

Adolescents reported their perception of parents’ aspirations for their education (“How far do you think your mother and father would like you to go in school?”) on a 6-point scale ranging from 1 = *completion of middle school* to 6 = *doctoral degree*. Similar measures used in previous research have shown good validity (e.g., Wu et al., 2018).

#### Close friends’ aspirations

Adolescents responded to one item regarding their close friends’ aspirations for their academic future (“To your knowledge, what is the highest level of education that your close friends would like to achieve?”). Responses were made on a 6-point scale ranging from 1 = *completion of middle school* to 6 = *doctoral degree*. Similar measures used in previous research have shown good validity (e.g., Hayes et al., 2015).

#### Attachment relationships with parents and peers

Adolescents’ perceptions of the quality of their relationships with their mothers, fathers, and peers were assessed using the Inventory of Parent and Peer Attachment (IPPA; Armsden & Greenberg, 1987). Paternal, maternal, and peer attachments were measured separately on a 25-item scale featuring three dimensions: trust (e.g., “When I am angry about something, my mother tries to be understanding”), communication (e.g., “My mother encourages me to talk about my difficulties”), and alienation/anger (e.g., “I get upset easily around my mother”). Responses were scored on a 5-point scale ranging from 1 = *almost never or never true* to 5 = *almost always or always true*. The IPPA has been shown to be a reliable and valid attachment measure for Chinese adolescents (Song et al., 2009). Alphas for maternal, paternal, and peer attachment scales ranged from 0.90 to 0.93.

### Analysis

Descriptive statistics were used to address the first hypothesis. To address the second hypothesis, structural equation modeling (SEM) with Amos 21.0 software (IBM SPSS, 2012) was used to test hypothetical models predicting pathways from individual, family, and peer factors to adolescent educational aspirations. In Step 1, confirmatory factor analysis (CFA) was carried out to evaluate the measurement model (Brown, 2006). In Step 2, SEM analysis using maximum likelihood estimation was applied to estimate the model fit and the path coefficients of the hypothesized models. The model fit was evaluated by the following indices: the chi-square fit index (χ^2^), the comparative fit index (CFI), the goodness-of-fit index (GFI), the adjusted GFI (AGFI), the Tucker‒Lewis index (TLI), and the root mean square error of approximation (RMSEA). The model fit is considered adequate when the coefficients of CFI, GFI, AGFI, and TLI are all greater than 0.90 (Bentler, 1983). For RMSEA, a coefficient less than 0.08 indicates an acceptable fit (Browne & Cudeck, 1993).

The hypothesized pathways from individual, family, and peer factors to educational aspirations were estimated, including direct pathways from each variable (i.e., family SES, other’s aspirations, attachments, and individual factors) to educational aspirations and separate indirect pathways from family SES, other’s aspirations, and parent and peer relationships to educational aspirations through individual factors. To examine the indirect effects, 2000 bootstrapped samples with 95% biased-corrected and percentile confidence intervals (CIs) were estimated (Taylor et al., 2008). Of the total sample, 78 students (12.9%) were missing data for maternal education level, and 81 (13.4%) were missing data for paternal education level. The Bayesian imputation method was used throughout the analysis to address missing data because of this method’s ability to improve statistical power (Buhi et al., 2008). The final dataset contained responses from 606 students.

## Results

### Educational aspirations of rural Chinese adolescents

The participants demonstrated high aspirations, with over 90% expressing a desire to obtain a higher education qualification. In our sample, 245 (40.4%) aspired to obtain a bachelor’s degree, 168 (27.7%) aspired to obtain a master’s degree, and 145 (23.9%) aspired to obtain a doctoral degree. 39 (6.4%) and eight (1.3%) had the goal of completing high school and vocational school, respectively. Only one participant reported no desire to continue his education beyond middle school.

### SEM preliminary analyses

Descriptive statistics, skewness, kurtosis, and intercorrelations between the main study variables are presented in Table 2. The distribution of the observed variables were considered to be univariate normal as all of the absolute values of the skewness (range: − 1.32–0.94) and kurtosis (range: − 1.15–2.34) indices were less than 3 and 8, respectively (Kline, 2005, p. 50). However, Marida’s normalized estimate of multivariate kurtosis for the educational aspirations model was 55.22 (> 5.00), indicating a violation of multivariate normality (Byrne, 2010). Therefore, bootstrapping was used to assess the approximate model fit indices and parameter estimates (Bollen & Stine, 1992). The correlations between the latent constructs ranged from 0.10 to 0.54, indicating the absence of multicollinearity (Grewal et al., 2004).

**Table 2:** Descriptive statistics and correlations of the main study variables (*N* = 606)

	1	2	3	4	5	6	7	8	9	10	11	12	13	14	15
1. Educational aspirations	–														
2. Academic self-regulation	.29^**^	–													
3. Goal valuation	.32^**^	.65^**^	–												
4. Attitudes toward teachers	.14^**^	.60^**^	.46^**^	–											
5. Academic self-perception	.26^**^	.72^**^	.35^**^	.51^**^	–										
6. Academic performance	.52^**^	.44^**^	.35^**^	.25^**^	.46^**^	–									
7. Mother's aspirations	.60^**^	.16^**^	.20^**^	.11^**^	.18^**^	.35^**^	–								
8. Father's aspirations	.58^**^	.19^**^	.20^**^	.13^**^	.18^**^	.35^**^	.77^**^	–							
9. Friends' aspirations	.51^**^	.18^**^	.21^**^	.14^**^	.12^**^	.26^**^	.42^**^	.42^**^	–						
10. Maternal attachment	.13^**^	.35^**^	.26^**^	.38^**^	.33^**^	.07	.04	.06	.04	–					
11. Paternal attachment	.07	.35^**^	.22^**^	.38^**^	.37^**^	.08^*^	.02	.06	.05	.53^**^	–				
12. Peer attachment	.16^**^	.18^**^	.15^**^	.32^**^	.19^**^	.10^*^	.15^**^	.15^**^	.13^**^	.29^**^	.26^**^	–			
13. Mother's educational level	.08	.04	.02	.04	.09^*^	.07	.09^*^	.08^*^	.05	.05	- .01	.03	–		
14. Father's educational level	.05	.10^*^	.09^*^	.04	.08	.03	.04	.08^*^	.04	.06	.06	.03	.41^**^	–	
15. Family affluence	.11^**^	.15^**^	.13^**^	.12^**^	.13^**^	.07	.05	.08^*^	.07	.24^**^	.17^**^	.12^**^	.24^**^	.23^**^	–
*M*	4.66	47.70	34.76	35.75	27.03	3.00	4.73	4.73	4.57	79.45	78.36	90.31	3.22	3.43	3.74
*SD*	0.97	11.64	6.49	8.13	8.35	1.33	0.91	0.91	0.99	18.01	19.10	15.99	1.05	1.12	1.60
Skewness	-0.17	-0.57	-1.32	-0.68	-0.10	0.06	0.01	0.01	- 0.24	-0.09	-0.06	-0.18	0.94	0.67	-0.08
Kurtosis	-0.35	0.18	2.34	0.64	-0.29	-1.15	-0.59	-0.76	0.41	-0.30	-0.37	-0.39	0.50	-0.14	-0.72

*p < .05; **p < .01

Confirmatory factor analysis (CFA) was used to assess the internal reliability, convergent validity, and discriminant validity of the four constructs in our proposed structural model (i.e., individual factors, others’ aspirations, attachments, and SES). The results indicated that the composite reliability (*CR*) of each construct ranged from 0.57 to 0.83, which exceeded the *CR* threshold value (0.6) and provided evidence of internal reliability (Bagozzi & Yi, 1989). The factor loadings for the individual items were all significant, providing evidence for the convergent validity of each measurement model. The average variance extracted (*AVE*) values for the constructs were acceptable (range: 0.32–0.59), as suggested by Fornell and Larcker (1981). Table 3 shows that the intercorrelations of all constructs were smaller than the square root of the *AVE* of each construct; thus, the level of discriminant validity was acceptable (Hair et al., 2006).

**Table 3:** Discriminant validity

	*AVE*	Individual factors	Others’ aspirations	Attachments	SES
Individual factors	0.506	**0.711**			
Others’ aspirations	0.589	0.256	**0.767**		
Attachments	0.407	0.543	0.103	**0.638**	
SES	0.319	0.163	0.143	0.173	**0.565**

Square root of *AVE* in bold on diagonals; Off diagonals are Pearson correlations of constructs *AVE* = Average variance extracted; SES = Socioeconomic status

### Structural model

**Fig. 1:**
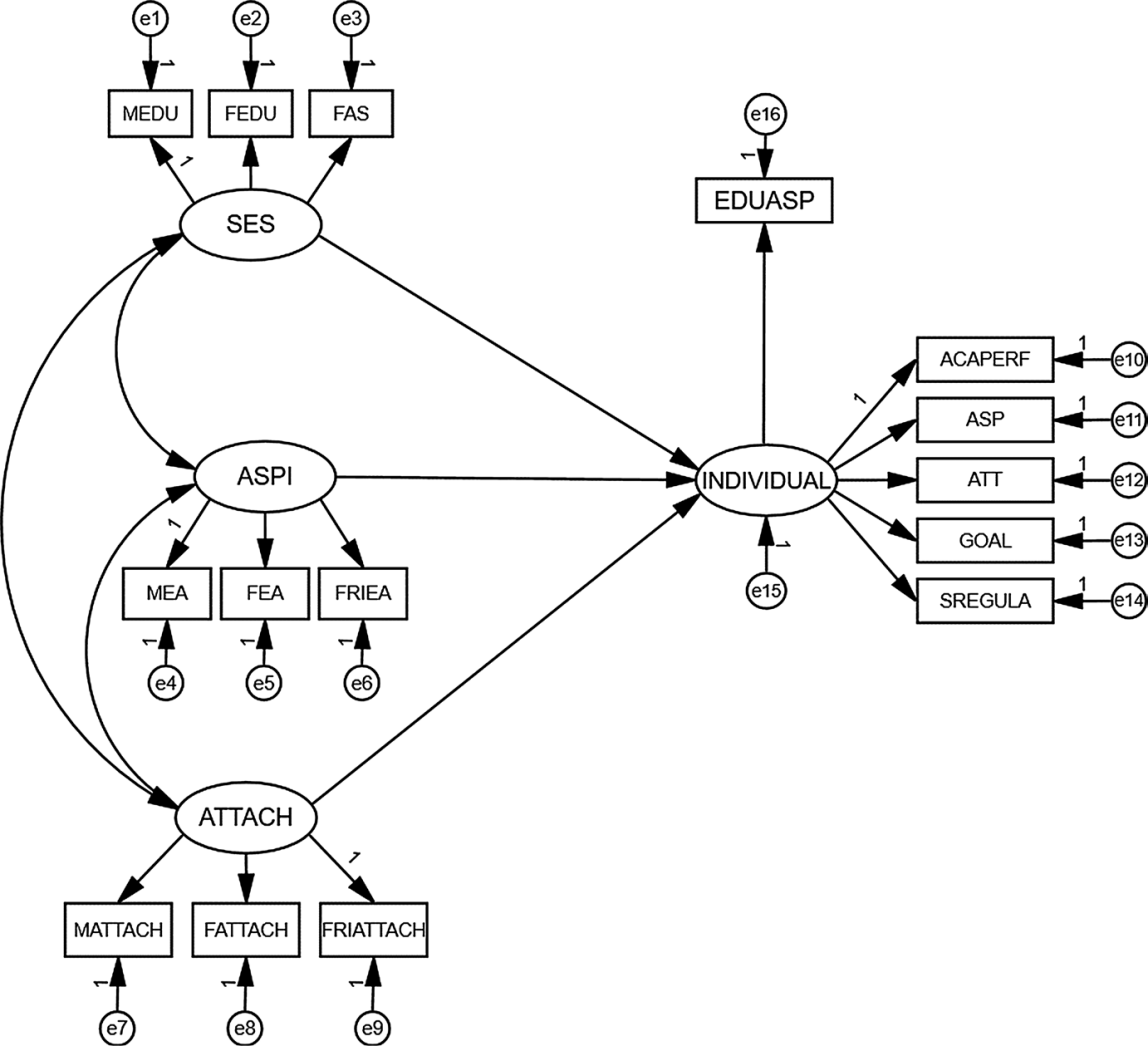
Hypothesized educational aspirations model. *Note.* Latent variable: SES = Family socioeconomic status; ASPI = Others’ aspirations; ATTACH = Perceived attachments to parents and peers; INDIVIDUAL = Individual factors. Observed variables: EDUASP = Educational aspirations; MEDU = Mother’s educational level; FEDU = Father’s educational level; FAS = Family wealth; MEA = Mother’s aspirations for children’s education; FEA = Father’s aspirations for children’s education; FRIEA = Close friends’ aspirations; MATTACH = The quality of attachment relationship with mother; FATTACH = The quality of attachment relationship with father; FRIATTACH = The quality of attachment relationship with peers; ACAPERF = Academic performance; ASP = Academic self-perception; ATT = Attitudes toward teachers; Goal = Goal valuation; SREGULA = Academic self-regulation

Figure 1 presents a hypothesized model for examining the factors associated with adolescents’ educational aspirations. The individual factors was a latent variable including five indicators: academic performance, academic self-perceptions, attitudes toward teachers, goal valuation, and academic self-regulation. The remaining three latent variables were family SES, others’ aspirations, and attachments. Mother’s educational level, father’s educational level, and self-perception of family affluence served as indicators of SES. The factor assessing others’ aspirations consisted of three indicators: mother’s aspirations for children, father’s aspirations for children, and the aspirations of close friends. Finally, the quality of adolescents’ relationships was indexed by items assessing maternal, paternal, and peer attachments. These three exogenous variables (SES, others’ aspirations, and attachments) were allowed to covary with each other.

**Fig. 2:**
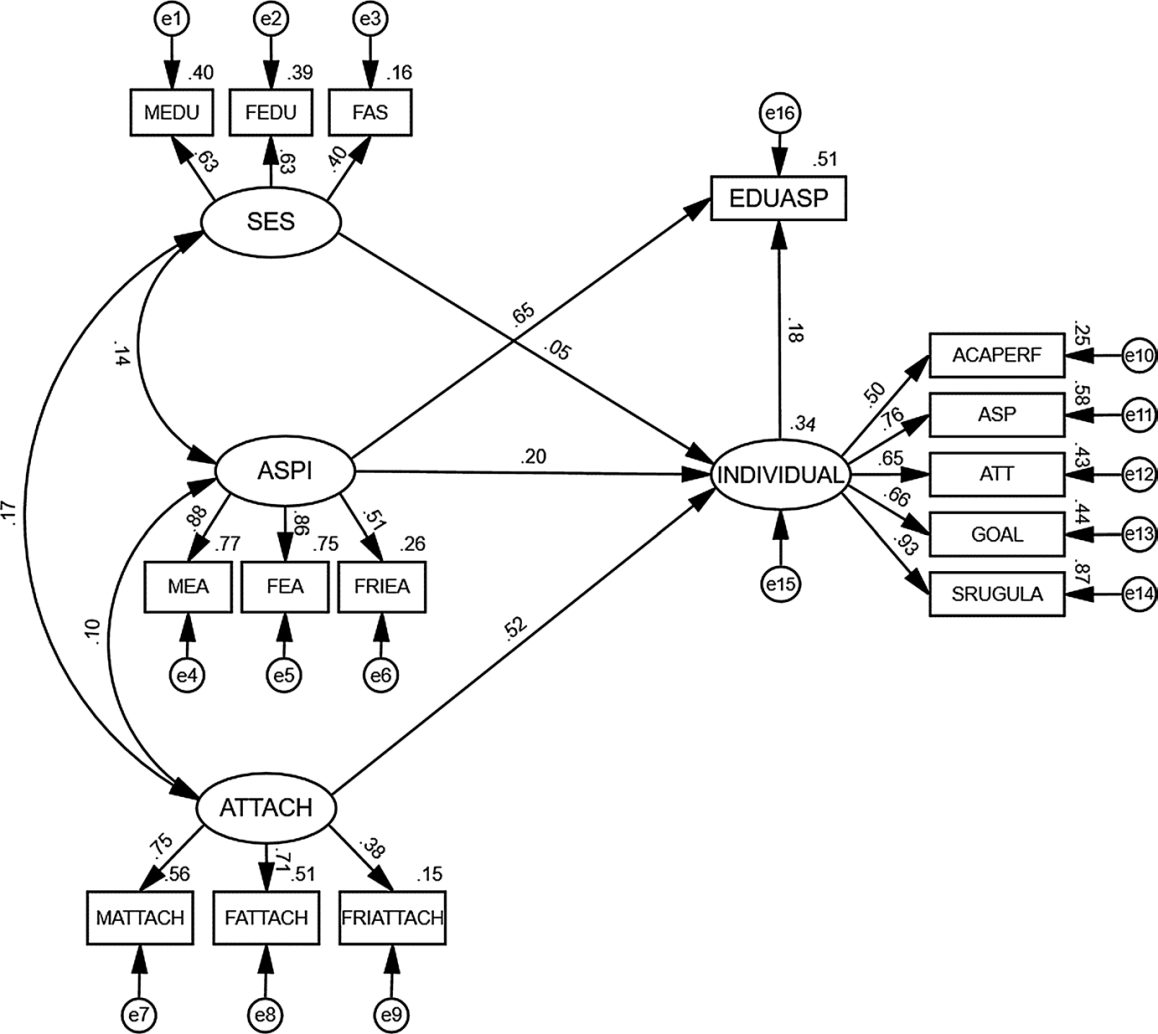
Modified educational aspirations model. *Note.* Latent variable: SES = Family socioeconomic status; ASPI = Others’ aspirations; ATTACH = Perceived attachments to parents and peers; INDIVIDUAL = Individual factors. Observed variables: EDUASP = Educational aspirations; MEDU = Mother’s educational level; FEDU = Father’s educational level; FAS = Family wealth; MEA = Mother’s aspirations for children’s education; FEA = Father’s aspirations for children’s education; FRIEA = Close friends’ aspirations; MATTACH = The quality of attachment relationship with mother; FATTACH = The quality of attachment relationship with father; FRIATTACH = The quality of attachment relationship with peers; ACAPERF = Academic performance; ASP = Academic self-perception; ATT = Attitudes toward teachers; Goal = Goal valuation; SREGULA = Academic self-regulation

The initial results of the hypothesized model showed a failure of convergence, χ^2^ (84, *N* = 606) = 762.85, *p* < .001, CFI = 0.79, GFI = 0.86, AGFI = 0.80, RMSEA = 0.12. Accordingly, the model was modified to include others’ aspirations as exerting a direct effect on educational aspirations to see if this change improved the model fit (see Fig. 2). Due to multivariate nonnormality, the Bollen–Stine *p* value correction was employed to correct for bias in the model fit statistics. The modified model fit the data well: χ^2^/df = 1.17; CFI = 0.99, GFI = 0.97, AGFI = 0.95, TLI = 0.99; RMSEA = 0.02. The results indicated that 51% of the variance in adolescents’ educational aspirations was explained by the modified model.

### Direct and indirect effects

The direct effects of both individual factors (β = 0.18, *b* = 0.26, *p* < .001) and others’ aspirations (β = 0.65, *b* = 0.78, *p* < .001) on adolescents’ educational aspirations were significant (see Table 4). Family SES and parents and peer attachments did not have a significant direct effect on adolescents’ educational aspirations. Of the five individual factors, academic self-regulation had the largest effect (β = 0.93, *p* < .001), followed by self-perception, goal valuation, attitudes toward teachers, and academic performance (β = 0.58, 0.44, 0.43, and 0.25, respectively, all *p*s < .001). In terms of the influence of others’ aspirations, mothers’ aspirations (β = 0.88, *p* < .001), followed by fathers’ aspirations (β = 0.86, *p* < .001), were more influential than close friends’ aspirations (β = 0.51, *p* < .001).

**Table 4:** Unstandardized total, indirect, and direct effects of the educational aspiration model

		Unstandardized coefficients (*b*)		Product of coefficients		Bootstrapping		Two-tailed significance
				Bias-corrected 95% CI		Percentile 95% CI		
		*SE*	Z	Lower	Upper	Lower	Upper	
*Unstandardized total effects*								
SES → Educational aspirations	0.019	0.064	0.295	− 0.106	0.149	− 0.106	0.149	.768
Others’ aspirations → Educational aspirations	0.825	0.055	14.919	0.724	0.950	0.721	0.948	< .001
Attachments → Educational aspirations	0.015	0.006	2.261	0.003	0.029	0.003	0.028	.024^*^
Individual factors → Educational aspirations	0.257	0.069	3.744	0.120	0.388	0.130	0.401	< .001
*Unstandardized indirect effects*								
SES → Individual factors → Educational aspirations	0.012	0.015	0.816	− 0.013	0.045	− 0.015	0.043	.414
Others’ aspirations → Individual factors → Educational aspirations	0.043	0.015	2.808	0.018	0.079	0.017	0.076	.005^**^
Attachments → Individual factors → Educational aspirations	0.014	0.005	3.015	0.007	0.025	0.007	0.026	.003^**^
*Unstandardized direct effects*								
SES → Educational aspirations	0.007	0.063	0.110	− 0.118	0.138	− 0.119	0.137	.913
Others’ aspirations → Educational aspirations	0.782	0.057	13.751	0.680	0.911	0.677	0.904	< .001
Attachments → Educational aspirations	< 0.001	0.008	0.034	− 0.015	0.017	− 0.016	0.015	.973
Individual factors → Educational aspirations	0.257	0.069	3.744	0.120	0.388	0.130	0.401	< .001

Unstandardized estimation of 2000 bootstrap samples

^*^p < .05; **p < .01

To investigate the indirect effects of contextual factors (SES, others’ aspirations, attachments) via individual factors on educational aspirations, we estimated bias-corrected percentiles at a 95% confidence interval with a bootstrapped sample of 2,000 (Taylor et al., 2008). There was a significant indirect effect of individual factors on the relationship between others’ aspirations (β = 0.036, *b* = 0.043, *p* < .01), attachments (β = 0.094, *b* = 0.014, *p* < .01) and educational aspirations (see Table 4). However, there was no significant indirect effect of individual factors on the relationship between SES and educational aspirations. There was a significant direct and indirect effect of others’ aspirations on educational aspirations, indicating partial mediation through individual factors. Attachment quality did not have a significant direct effect on aspirations but had a significant indirect effect on aspirations through individual factors.

## Discussion

In the current study, adolescents in rural China reported high educational aspirations (mean score of 4.66 and maximum score of 6), with more than 92% planning to pursue higher education. An overall model including individual and contextual factors (i.e., family SES, others’ aspirations, and perceived attachments to parents and peers) explained the educational aspirations of these adolescents, which is consistent with previous research conducted in Western countries (e.g., Garg et al., 2002, 2007; Hartas, 2016). Importantly, by highlighting the separate and cumulative contributions of individual and contextual factors in shaping the educational aspirations of rural Chinese youth, the results provided support for ecological and SCC models of developmental competencies in non-Western samples.

Our study found that individual factors, including academic performance, academic self-perceptions, academic self-regulation, attitudes toward teachers, and goal valuation, had a significant direct effect on adolescents’ educational aspirations. This result supports the critical role of cognitive-person variables in influencing student goal setting, in line with SCCT (Lent et al., 2000). This finding is also consistent with previous research that links students’ positive feelings about their ability to perform a task successfully and the value of the goal to their goal setting and actions (Curtin et al., 2016; Guo et al., 2015). In addition, we found the strongest effect of academic self-regulation on aspirations among the variables defining individual factors. Thus, students who are skilled and actively involved in managing their learning are likely to complete academic tasks at a high standard, thereby promoting their confidence in achieving their educational goals (De la Fuente et al., 2015). The current findings suggest that theoretical models would benefit from highlighting self-regulation as an important contributor to educational aspirations.

Others’ aspirations also had a direct effect on adolescents’ educational aspirations and explained the most variance (42.3%) in adolescents’ educational aspirations of all the factors included in the model. Parents’ aspirations appeared to be more strongly related to adolescents’ educational aspirations in our study than in studies of adolescents in Western nations (e.g., Garg et al., 2002). There are several possible explanations for this finding. First, individualistic cultures in the Western context usually encourage children to rely less on their parents, which leads adolescents to have a more independent view of their future (Kitayama & Uskul, 2011); however, collectivist cultures such as China place a high value on children’s obedience and respect for their parents (Luo et al., 2013). Moreover, East Asian parents are more likely to emphasize education for their children than their Western counterparts (Park et al., 2011). Therefore, our results suggest that parents’ beliefs and values concerning the pursuit of higher education have an important influence on the educational goals of rural Chinese adolescents.

The current findings indicated that although the relative influence of close friends’ aspirations on adolescents’ educational aspirations was lower than that of perceived parents’ aspirations, close friends were also found to be a significant source of inspiration for rural adolescents. This finding is consistent with previous studies indicating that adolescents tend to make decisions similar to those of their close friends concerning future educational trajectories (Hemi et al., 2023; Sokatch, 2006), which may reflect the prominence of peer influence in early adolescence (Ryan, 2001). Considering SCCT’s proposition (Lent et al., 2000) that contextual influences can affect people’s academic development both directly and indirectly, our finding that others’ aspirations have a direct influence on adolescents’ aspirations provides a more nuanced understanding of the important role that significant people in the immediate context (i.e., family and school) play in influencing adolescents’ academic decisions in a social context that places a high value on connectedness. Thus, our findings suggest that policies and interventions aimed at promoting the educational aspirations of rural Chinese adolescents should target peer and family processes.

Perceptions of significant others’ aspirations were also indirectly related to educational aspirations through individual factors, which is consistent with previous findings in Western samples (Garg et al., 2002; Hemi et al., 2023). Parental educational aspirations for their children have been identified as the strongest predictor of children’s academic self-efficacy (Yamamoto & Holloway, 2010). Similarly, secondary school students’ perceived peer achievement goals are directly related to academic motivation (Jiang et al., 2014). Consistent with these studies, our findings suggest that members of family and peer groups characterized by high aspirations promote a positive sociocognitive process in the school environment, which in turn promotes high aspirations among adolescents living in rural China.

Similarly, our findings highlight the promotive role of positive parent and close friend relationships in education, with individual factors fully mediating the relationship between attachments and adolescents’ educational aspirations. Consistent with SCCT and previous research (Moss & St-Laurent, 2001), high-quality attachment relationships with parents and peers may serve as a secure foundation for independent learning and exploration, thereby increasing adolescents’ academic motivation and perceived competence, which in turn leads adolescents to hold higher educational aspirations.

Altogether, the indirect effects of others’ aspirations and attachments on educational aspirations through individual factors highlight the constant interaction between the individual and his or her environment (Bronfenbrenner, 1979; Garbarino & Ganel, 2000). Moreover, consistent with previous research inspired by SCCT (e.g., Lent et al., 2000), our findings also suggest that adolescents are more likely to pursue their academic and career goals when they perceive these goals to be driven by favorable contextual factors (e.g., secure attachments and their immediate social circle holding high aspirations). Importantly, our findings provide support for the application of ecological and SCCT frameworks to the educational context of rural China.

One unexpected result was that family SES did not have a significant effect on adolescents’ aspirations. However, this finding contrasts with the results of Western studies, where show that adolescents from a high SES background are more likely to have higher educational aspirations than those from socially disadvantaged backgrounds (Garg et al., 2002; Lee & Byun, 2019). There are several possible explanations for these inconsistent findings. First, our study sample was fairly homogeneous in terms of SES, with only a small number of adolescents coming from high SES backgrounds (i.e., family affluence was categorized as “high” and parents obtained higher education). The second explanation is related to East‒West cultural differences. In Western countries, such as the USA and the United Kingdom, students from high-SES families are more likely to have access to higher education than their lower-SES counterparts with similar test scores or grades (Destin & Oyserman et al., 2009; Jackson et al., 2007). Furthermore, due to the wide gap between rich and poor in the USA (Flores, 2017), students from high-SES households are more likely to aspire to higher education than low-SES students who are more concerned about their career goals (Browman et al., 2022). Thus, in Western countries, family SES may be more important than academic performance in relation to students’ educational choices. In contrast, Chinese culture emphasizes that success is achieved through diligence and self-effort (Li, 2003). As a result, Chinese students tend to attribute their educational success to their academic ability rather than external factors (Sun et al., 2021). Thus, it is possible that the emphasis on education in China has contributed to rural adolescents’ desire for education regardless of their family SES.

It is important to acknowledge the limitations of the current study when interpreting our findings. First, we relied on youth self-reports of individual, peer, and family factors; thus, it is possible that relationship inflation may have resulted from shared method variance. While young people are best placed to report their own educational aspirations, they may not accurately report the aspirations of their parents and peers. The current findings refer to adolescents’ perceptions of others’ educational aspirations. Future research should include parent and peer reports to increase our understanding of the degree to which self- versus other-perceptions of educational aspirations are important to adolescents’ educational aspirations. Second, the outbreak of COVID-19 severely affected researchers’ ability to collect data from different provinces. Although Hubei province is a suitable setting for studying rural Chinese youth, the current study was limited by the fact that it only surveyed students from two schools in a specific rural township, thus limiting the generalizability of the findings. Future research should sample across schools from a broader range of rural areas in China. Third, the current study was cross-sectional, which prevented us from making causal inferences and examining potential reciprocal relationships between the main study variables. However, given the absence of prior research examining the pathways between individual and contextual factors and educational aspirations in rural Chinese adolescents, this initial research using a cross-sectional design provides useful information to guide more time- and resource-intensive longitudinal research in the future. Finally, considering the relative paucity of validated scales measuring educational aspirations in the literature (Leung et al., 2017), the current study is consistent with most previous research in using a single item to assess adolescents’ educational aspirations. Future research may wish to compare the validity of single and multi-item scales assessing educational aspirations in East Asian nations.

## Conclusion and implications

The current study provides support for Bronfenbrenner’s (1979) ecological theory and Lent et al.’s (2000) SCCT as frameworks to enhance our understanding of the nature of educational aspirations in Chinese adolescents in rural areas and the pathways via which individual and contextual factors influence these goals. The findings indicate that the examined young people place a high value on education and that individual factors (i.e., attributes related to adolescents' internal cognitive and affective states in the school domain), as well as the aspirations of parents and friends, play critical roles in shaping their educational aspirations. An important implication of these findings is that parents should be encouraged to discuss future educational goals with their children in early adolescence; moreover, a close and supportive parent‒child relationship is needed to facilitate good communication. Family-based intervention programs may be useful to help parents develop close, confiding relationships with their teenage children. School-based programs may also assist in not only fostering good communication with parents, adolescents, and teachers but also in helping rural adolescents develop supportive peer relationships. Furthermore, promoting a sense of school environment within peer groups is likely to boost adolescents’ educational aspirations. For example, young people could lead regular activities and group discussions about their future educational and career goals and the different pathways available to realize these aspirations. Future research should track individual, family, and peer influences from early adolescence to young adulthood to increase our understanding of how these factors evolve over time in terms of their interrelationships with Chinese adolescents’ educational aspirations across the transition from middle to senior school to help further education.

## Data Availability

The data that support the findings of this study are available on request from the corresponding author. The data are not publicly available due to privacy or ethical restrictions.
